# Exploring the Antibacterial and Antibiofilm Properties of Lactic Acid Bacteria Isolated From Sucuk and Dairy Products

**DOI:** 10.1002/vms3.71102

**Published:** 2026-07-21

**Authors:** Neslihan Öztürk, Hatice Ahu Kahraman, Erhan Keyvan, Erdi Şen

**Affiliations:** ^1^ Institute of Health Sciences Burdur Mehmet Akif Ersoy University Burdur Turkey; ^2^ Department of Food Hygiene and Technology Faculty of Veterinary Medicine Burdur Mehmet Akif Ersoy University Burdur Turkey

**Keywords:** antibiofilm activity, artisanal dairy and meat, cell‐free supernatants, lactic acid bacteria, sucuk

## Abstract

**Background:**

Lactic acid bacteria (LAB) isolated from traditional artisanal products without starter cultures represent a valuable source of novel strains with high potential.

**Objectives:**

This study aimed to investigate the antibacterial and antibiofilm effects, as well as antibiotic resistance, of LAB isolated from traditional artisanal dairy and meat products.

**Methods:**

LAB identification was performed using MALDI‐TOF‐MS and 16S rRNA gene sequencing. The antimicrobial and antibiofilm activity of LAB isolates was assessed against *Staphylococcus aureus* (ATCC 25923), *Listeria monocytogenes* (ATCC 13932), *Escherichia coli* (ATCC 29998) and *Bacillus cereus* (ATCC 11778).

**Results:**

A total of 90 isolates were obtained from 45 samples, and 35 isolates confirmed as LAB were further characterized. Out of the 35 LAB isolates, 7 demonstrated selective inhibition zones against *L. monocytogenes* and *B. cereus*, while showing no inhibition against *S. aureus* and *E. coli*. All seven isolates were found to be susceptible to chloramphenicol and ampicillin, and intermediately susceptible to gentamicin. Moreover, 42.85% of the isolates were phenotypically resistant to trimethoprim‐sulfamethoxazole and 57.14% to ciprofloxacin and oxacillin. Among the seven potent strains, six showed significant antibiofilm activity (85%–100%) against at least one of the pathogens tested. The six dual‐action isolates were later identified by 16S rRNA gene sequencing as *Pediococcus pentosaceus* (I‐3, I‐21 and I‐36) and *Lactiplantibacillus plantarum* (I‐5, I‐6 and I‐34), with 100% similarity to the reference sequences.

**Conclusions:**

These results highlight the potential of traditional artisanal strains as natural antimicrobials and antibiofilm agents, which are contributing to ongoing evaluations of protective cultures in the health, food and biotechnology fields.

## Graphical abstract

Lactic acid bacteria (LAB) were isolated and characterized for their antibiotic resistance and safety profiles, as well as their potent antibacterial and antibiofilm activities against pathogens. The most effective strains were successfully identified via 16S rRNA gene sequencing for future functional applications.

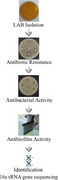

## 1 Introduction

Lactic acid bacteria (LAB) are bacteria that produce lactic acid through fermentation, giving products taste, smell and aroma. Traditional fermented foods represent a rich source of LAB. Among fermented foods, traditional meat and dairy products are considered the most important sources of probiotic LAB (Zago et al. 2011). LAB contain a significant number of bacterial species, such as *Lactobacilli*, *Lactococci*, *Streptococci*, *Leuconostoc* and *Pediococci*, which are used in industrial applications. LAB are found in many environments, such as fermented dairy products, meat, fish, sourdough and pickles, as well as in the healthy intestinal microbiota of humans and animals. In addition, probiotic species compete with pathogens in the intestinal microflora and stimulate mucosal immunity (Klaenhammer et al. 2002).

Foodborne pathogens pose significant public health problems worldwide. Common foodborne bacterial pathogens include *Listeria monocytogenes, Escherichia coli* O157:H7, *Staphylococcus aureus*, *Salmonella* Enteritidis, *Bacillus cereus*, *Vibrio* spp., *Campylobacter jejuni*, *Clostridium perfringens* and Shiga toxin‐producing *E. coli* (Law et al. 2015). Foodborne pathogens cause foodborne illnesses, also known as foodborne infections and food poisoning. According to the Centers for Disease Control and Prevention (CDC), more than 250 pathogens and toxins are known to cause foodborne diseases (CDC 2024). LAB have shown significant interest in food biotechnology for its ability to prevent the growth of pathogenic bacteria, thereby eliminating the risks associated with these pathogens (Cirat et al. 2024). They produce a wide range of antimicrobial substances, such as lactic acid, acetic acid, ammonia, bacteriocins, ethanol, reuterin, hydrogen peroxide and diacetyl, which inhibit the development of foodborne illnesses, suppress food spoilage and impede the growth of pathogenic microorganisms (Cirat et al. 2024; Li et al. 2026). Additionally, LAB contribute to food preservation by delaying spoilage, acting as natural food preservatives and enhancing the development of flavour and aroma in food products (Shi and Maktabdar 2022). The formation of biofilm layers typically begins with the attachment and proliferation of bacteria to organic material that accumulates on equipment, tools and surfaces used in production and processing lines. The biofilm layer enables microorganisms to withstand osmotic stress, toxic compounds, disinfectants and antibiotics. Biofilm formation is closely associated with pathogenicity and directly linked to the antibiotic resistance of pathogenic microorganisms (Turhan and Erginkaya 2019). Therefore, controlling the important pathogenic microorganisms of public health concern should involve strategies to prevent biofilm formation.

Highly diverse microbial communities are observed in traditional, artisanal fermented foods and, unlike in industrial food production, rely solely on natural microbiota (Marco et al. 2021). Robust LAB populations are naturally selected by environmental stresses present throughout traditional processing (Tamang et al. 2016). For isolating possibly novel probiotic and bioprotective LAB strains with specific technical features and strong antibacterial and antibiofilm properties, these conventional matrices are considered highly significant and promising resources (Marco et al. 2021; Leroy and De Vuyst 2004). This study aims to isolate LAB from various traditional artisanal dairy and meat products and investigate their antimicrobial and antibiofilm effects on significant foodborne pathogens.

## 2 Materials and Methods

### 2.1 Samples

Different food products produced traditionally without the use of any commercial starter cultures were obtained from various bazaars in Burdur Province of Turkey, including raw cow milk (*n* = 3), yogurt (*n* = 3), butter (*n* = 12), white cheese (*n* = 10), Tulum cheese (*n* = 3), milk crema (*n* = 5) and Turkish dried‐fermented sucuk (*n* = 9). The food samples were aseptically collected in single‐use sterile sampling containers, maintaining the cold chain, and transported to the laboratory. The isolation process was begun on the same day, without storage.

### 2.2 Isolation of the LAB Cultures

Ten grams of the sample were mixed thoroughly with 90 mL of 0.1% peptone water (Merck, Darmstadt, Germany), and dilutions were prepared. A total of 100 µL of each dilution was spread onto De Man, Rogosa and Sharpe agar (MRS, Merck, Darmstadt, Germany) and then incubated anaerobically at 30°C for 48 h. After incubation, two colonies from each sample with distinct morphological characteristics (such as colour, consistency, shape, size and edge type) were selected and purified by streaking and subculturing on MRS agar. The isolated colonies were subjected to Gram staining, catalase testing and haemolysis testing. Of the 90 isolates, 35 were identified as Gram‐positive, non‐haemolytic and catalase‐negative. The purity of the isolates was evaluated under a microscope. The LAB isolates were stored in MRS broth containing 20% glycerol at −20°C (Kamiloğlu 2022).

### 2.3 Identification of LAB

Isolates were identified by MALDI‐TOF MS using the previously described method (Uysal et al. 2019). Briefly, individual fresh colonies were suspended in 300 µL of sterile distilled water, and 900 µL of ethanol was added. The bacterial suspension was then centrifuged at 9795 × *g* for 5 min at room temperature (Eppendorf 5424R, Germany), and the supernatant was discarded. The pellet was resuspended in a mixture of 50 µL formic acid (70%) and 50 µL acetonitrile. After centrifugation at 9795 × *g*, 20°C for 3 min, 1 µL of the supernatant was placed onto a steel target plate and allowed to air‐dry. Each spot was covered with 1 µL of a matrix solution containing α‐CHCA. Once dried, the samples were analysed using MALDI Biotyper (Biotyper 3.0; Microflex LT, Bruker Daltonics GmbH, Bremen, Germany) running with the BioTyper database version DB‐5989, containing 5989 reference MALDI‐TOF MS profiles. The test standard provided by Bruker Daltonics was used according to the manufacturer's instructions.

### 2.4 Antibacterial Activity of Cell‐Free Supernatants

The antibacterial activity of the isolates was assessed using the agar well method with cell‐free culture supernatant (Gharib et al. 2020). The isolated lactic acid bacteria were inoculated into MRS broth (Merck, Darmstadt, Germany) and incubated at 37°C for 48 h in a CO_2_ incubator (Nüve, Ankara, Turkey). Cell‐free supernatants (CFSs) were obtained by centrifuging bacterial suspensions at 10,000 × g for 10 min at 4°C (Eppendorf 5424R, Germany). The CFS was then sterilized using a 0.22 µm pore size filter (Millipore Inc., Billerica, USA) and used immediately without pH neutralization. The aim was to evaluate the total antagonistic activity of the isolates by closely mimicking the natural food medium in which LAB lower the pH to inhibit pathogens. The antibacterial activity was assessed against *E. coli* (ATCC 29998), *L. monocytogenes* 4b (ATCC 13932), *S. aureus* (ATCC 25923) and *B. cereus* (ATCC 11778). The presence of a visible halo around the well indicated inhibitory activity by the isolates.

### 2.5 Resistance to Antibiotics

The disc diffusion test was performed according to the method described by Tumbarski et al. (2021) with slight modifications. Initially, all isolates were adjusted to a 0.5 McFarland standard and swab streaked onto MRS agar, then incubated at 37°C for 48 h. The isolates were then evaluated for their susceptibility to six antibiotics: ampicillin (10 µg), oxacillin (1 µg), trimethoprim‐sulfamethoxazole (25 µg), ciprofloxacin (5 µg), gentamicin (120 µg) and chloramphenicol (30 µg). Bacterial strains that displayed no inhibition zones were considered resistant, while inhibition zones ranging from 7 to 16 mm were classified as intermediate susceptibility and zones greater than 16 mm were categorized as susceptible (Tumbarski et al. 2021).

### 2.6 Antibiofilm Activity

Antibiofilm activity of LAB isolates was assessed as described by Thenmozhi et al. (2009). LAB isolates were inoculated into MRS broth and incubated at 37°C for 48 h. After incubation, the samples were centrifuged at 10,000 × *g* for 10 min at 4°C (Eppendorf 5424R, Germany), and the supernatants were collected by passing them through a 0.2 µm pore‐size membrane filter. The test pathogens were streaked onto Tryptic Soy agar (TSA, Merck, Darmstadt, Germany) and incubated at 37°C for 24 h. Following incubation, the cultures were adjusted to a 0.5 McFarland turbidity. Then, 100 µL of bacterial culture and 100 µL of the isolated strain's CFS were transferred to 96‐well plates and incubated at 37°C for 24 h. Alongside the test samples, enrofloxacin (40 µg/mL) was used as a positive antibiofilm control, untreated bacterial suspensions served as the negative control and sterile broth media were included as blank wells. After incubation, the liquid medium in the wells was discarded, and the plates were washed three times with sterile distilled water. They were then inverted and dried on absorbent paper. 150 µL of a 0.5% crystal violet solution was added to each well, and the plates were incubated at room temperature for 45 min. Subsequently, the wells were washed three times with distilled water, inverted and dried in a 50°C incubator. To dissolve the dye, 200 µL of glacial acetic acid (30%) was added to each well and allowed to sit for 10 min. Then, 125 µL eluted solution from each well was transferred to a new plate and read at 630 nm using the microplate reader (Epoch, BioTek, USA). The antibiofilm activity of the cell‐free supernatants was calculated based on the following equation (Sandberg et al. 2008):

% Inhibition = (ODcontrol − OD sample / OD control) × 100

### 2.7 Amplification of the 16S rRNA Region

Amplification was performed as described by Klindworth et al. (2013), with some modifications. The amplification targeted a specific region using two primers, namely 8F (5' AGAGTTTGATCMTGGCTCAG 3') and 1387R (5' GGGCGGWGTGTACAAGGC 3'). For DNA isolation and bacterial species determination, the EurX GeneMATRIX Bacterial & Yeast DNA isolation kit from Poland was used. The PCR analysis was carried out using 5× FIREPol Master Mix (Solis Biodyne, Tartu, Estonia) in a total volume of 20 µL, followed by pre‐denaturing at 95°C for 5 min, followed by 30 cycles of denaturation at 95°C for 45 s, annealing at 57°C for 45 s and extension at 72°C for 60 s. The final extension step at 72°C for 5 min marked the completion of the amplification procedure. The size of each amplicon was assessed by running the samples on a 1.5% agarose gel containing ethidium bromide.

### 2.8 Amplified DNA Extracts Sequencing

The amplified DNA extracts were sequenced using the ABI 3730XL Sanger platform (Applied Biosystems, Foster City, CA). The sequencing process utilized the BigDye Terminator v3.1 Cycle Sequencing Kit (Applied Biosystems, Foster City, CA). The concentration of the amplified DNA extracts was confirmed using Thermo Scientific NanoDrop 2000 (USA). The resulting sequences were compared to those in the NCBI database (http://www.ncbi.nlm.nih.gov) using BLAST for sequence alignment and identification.

### 2.9 Statistical Analysis

All examined parameters were tested in triplicate, and results are presented as mean ± standard deviation (M ± SD). The suitability of the data for a normal distribution was assessed by examining skewness and kurtosis. The skewness and kurtosis values were in the ideal range (between −1 and +1) accepted in the literature for each scale. A one‐way analysis of variance (ANOVA) and Duncan's multiple range test were used to determine significant differences (*p* < 0.05).

## 3 Results

### 3.1 Isolation and Identification of Bacterial Strains

Overall, 90 bacteria isolated from 45 traditional artisanal dairy and meat products were initially characterized based on their cultural, morphological and biochemical properties, and 35 isolates were confirmed as LAB. A total of 35 isolates identified MALDI‐TOF MS (Table 1) belonged to 8 bacterial genera (including *Leuconostoc*, *Levilactobacillus*, *Lactiplantibacillus*, *Lactilactobacillus*, *Lactococcus*, *Lacticaseibacillus*, *Pediococcus* and *Streptococcus*) and 11 species (including *Leuconostoc mesenteroides* [*n* = 9], *Lactiplantibacillus plantarum* [*n* = 6], *Latilactobacillus sakei* [*n* = 5], *Lactococcus lactis* [*n* = 5], *Pediococcus pentosaceus* [*n* = 3], *Lacticaseibacillus rhamnosus* [*n* = 2], *Lacticaseibacillus paracasei* [*n* = 1], *Latilactobacillus coryniformis* [*n* = 1], *Levilactobacillus brevis* [*n* = 1], *Streptococcus thermophilus* [*n* = 1] and *Streptococcus lutetiensis* [*n* = 1]).

**TABLE 1:** MALDI‐TOF/MS results of the isolates.

No	Isolate code	Product	Score	Best match
1	I‐1	White cheese	2.125	*Lacticaseibacillus paracasei*
2	I‐2	Yogurt	2.224	*Leuconostoc mesenteroides*
3	I‐3	White cheese	2.393	*Pediococcus pentosaceus*
4	I‐4	White cheese	2.393	*Lacticaseibacillus rhamnosus*
5	I‐5	White cheese	2.301	*Lactiplantibacillus plantarum*
6	I‐6	White cheese	2.415	*Lactiplantibacillus plantarum*
7	I‐8	White cheese	1.934	*Latilactobacillus coryniformis*
8	I‐9	White cheese	2.268	*Lactiplantibacillus plantarum*
9	I‐12	Raw milk	2.319	*Lactococcus lactis*
10	I‐13	Crema	2.377	*Lactococcus lactis*
11	I‐14	Butter	2.144	*Lactococcus lactis*
12	I‐16	Butter	2.278	*Streptococcus thermophilus*.
13	I‐17	Butter	2.174	*Streptococcus lutetiensis*
14	I‐18	Crema	2.274	*Lactococcus lactis*
15	I‐19	Crema	2.247	*Lactococcus lactis*
16	I‐20	White cheese	2.477	*Levilactobacillus brevis*
17	I‐21	Raw milk	1.981	*Pediococcus pentosaceus*
18	I‐24	Sucuk	2.224	*Leuconostoc mesenteroides*
19	I‐25	Crema	2.429	*Leuconostoc mesenteroides*
20	I‐26	Butter	2.403	*Leuconostoc mesenteroides*
21	I‐27	Tulum cheese	2.416	*Latilactobacillus sakei*
22	I‐28	Butter	2.449	*Latilactobacillus sakei*
23	I‐29	Raw milk	2.296	*Leuconostoc mesenteroides*
24	I‐31	Butter	2.430	*Latilactobacillus sakei*
25	I‐32	Butter	2.397	*Leuconostoc mesenteroides*
26	I‐33	Sucuk	2.343	*Latilactobacillus sakei*
27	I‐34	Sucuk	2.254	*Lactiplantibacillus plantarum*
28	I‐35	Butter	2.062	*Leuconostoc mesenteroides*
29	I‐36	Sucuk	2.312	*Pediococcus pentosaceus*
30	I‐37	Sucuk	2.349	*Latilactobacillus sakei*
31	I‐39	Sucuk	2.368	*Leuconostoc mesenteroides*
32	I‐41	Sucuk	2.032	*Latilactobacillus sakei*
33	I‐42	Tulum cheese	2.368	*Leuconostoc mesenteroides*
34	I‐43	Raw milk	2.063	*Lactiplantibacillus plantarum*
35	I‐45	White cheese	2.478	*Lacticaseibacillus rhamnosus*

### 3.2 Antimicrobial Activity of the Cell‐Free Supernatants

The antimicrobial efficacy of CFS from 35 isolates was evaluated against *L. monocytogenes, B. cereus, E. coli* and *S. aureus*. The inhibitory diameters (mm) of the isolates are presented in Table 2. The inhibition zone data obtained in this study include the 6 mm initial well diameter. Enrofloxacin (40 µg/mL) was employed as a positive control in the antimicrobial assessment. Among the LAB isolates, seven of them exhibited inhibitory activity against at least one of the tested foodborne pathogens. Specifically, six isolates (I‐3, I‐5, I‐6, I‐20, I‐21 and I‐34) displayed inhibition zones ranging from 8 to 11 mm against *L. monocytogenes*. Regarding *B. cereus*, five isolates (I‐3, I‐6, I‐21, I‐34 and I‐36) showed inhibition zones ranging from 8 to 10 mm. The highest inhibition rate against *L. monocytogenes* was observed in I‐20 and I‐21 (11 mm), while the lowest was observed in I‐3 (8 mm). Regarding *B. cereus*, the highest inhibition rate was observed in I‐6 (10 mm), while the lowest inhibition rate was observed in I‐3 (8 mm). Notably, none of the isolates exhibited antimicrobial activity (NI) against *S. aureus* and *E. coli* under the tested conditions. The remaining isolates did not show any inhibition zones against the tested pathogens. Further analyses were conducted with the seven isolates (I‐3, I‐5, I‐6, I‐20, I‐21, I‐34 and I‐36), all of which exhibited antibacterial properties.

**TABLE 2:** Antimicrobial activity of the isolated LAB strains against some pathogen bacteria (mm).

Isolate	*Staphylococcus aureus*	*Escherichia coli*	*Listeria monocytogenes*	*Bacillus cereus*
I‐3	NI	NI	8	8
I‐5	NI	NI	9	NI
I‐6	NI	NI	9	10
I‐20	NI	NI	11	NI
I‐21	NI	NI	11	10
I‐34	NI	NI	10	9
I‐36	NI	NI	NI	9
Enrofloxacin	25.15 ± 0.55	29.95 ± 0.87	22.65 ± 0.97	30.46 ± 0.64

*Note*: The results include the well diameter (6 mm). Enrofloxacin (40 µg/mL) was used as a positive control.

Abbreviation: NI—no inhibition.

### 3.3 Resistance to Antibiotics

The antibiotic resistance of isolated LAB was determined using the disc diffusion method, and the zone diameters were measured as presented in Table 3. In this study, zone measurements were performed for ampicillin (10 µg), gentamicin (120 µg), ciprofloxacin (5 µg), trimethoprim‐sulfamethoxazole (25 µg), oxacillin (1 µg) and chloramphenicol (30 µg). Based on the size of the inhibition zone, strains were classified as resistant (R) if they had no inhibition zone, intermediate (I) if the zone diameter ranged from 7 to 16 mm and susceptible (S) if the zone diameter was greater than 16 mm. According to the study results, all seven LAB isolates were susceptible (> 16 mm) to chloramphenicol (30 µg) and ampicillin (10 µg). All LAB isolates exhibited intermediate susceptibility (7–16 mm) to gentamicin (120 µg). Among the isolates, I‐20, I‐21 and I‐36 were resistant to trimethoprim‐sulfamethoxazole (25 µg), while the remaining isolates were susceptible (> 16 mm). The isolates I‐3, I‐5 and I‐20 were intermediate susceptible to Ciprofloxacin (5 µg), while the remaining isolates were resistant. Isolate I‐3, I‐5 and I‐34 exhibited intermediate susceptibility to Oxacillin (1 µg), whereas the remaining isolates were resistant.

**TABLE 3:** Results of antibiotic susceptibility tests of LAB isolates.

Isolate	Ampicillin (10 µg)	Gentamicin (120 µg)	Ciprofloxacin (5 µg)	Trimethoprim‐sulfamethoxazole (25 µg)	Oxacillin (1 µg)	Chloramphenicol (30 µg)
I‐3	30(S)	15(I)	16(I)	21(S)	10(I)	30(S)
I‐5	24(S)	16(I)	15(I)	21(S)	16(I)	32(S)
I‐6	25(S)	15(I)	(R)	18(S)	(R)	24(S)
I‐20	25(S)	12(I)	8(I)	(R)	(R)	25(S)
I‐21	19(S)	10(I)	(R)	(R)	(R)	25(S)
I‐34	28(S)	12(I)	(R)	22(S)	10(I)	24(S)
I‐36	19(S)	11(I)	(R)	(R)	(R)	25(S)

*Note*: R—resistant (no zone of inhibition / 0 mm zone diameter was evaluated as R); I—intermediate (zone 7–16 mm); S—susceptible (zone > 16 mm); disc = 6 mm.

### 3.4 Antibiofilm Effects of Isolates

**FIGURE 1:**
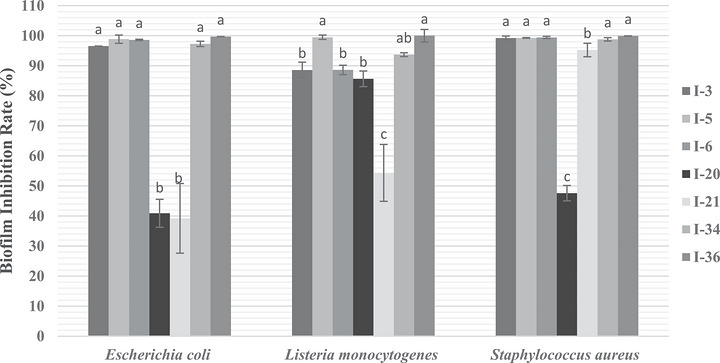
Antibiofilm effect (%) of isolates’ cell‐free supernatants against test pathogens. Results are represented as mean ± standard deviation (M±SD). Values with different letters (a–c) within a column differ significantly (*p* < 0.05).

The antibiofilm effects of cell‐free supernatants prepared from isolated LAB against *E. coli*, *L. monocytogenes* and *S. aureus*, known for their biofilm‐forming ability, were determined using the 96‐well microplate method, and the results are presented in Figure 1. It was observed that, except for I‐20 and I‐21, all five other isolates (I‐3, I‐5, I‐6, I‐34 and I‐36) exhibited antibiofilm effects ranging from 85% to 100%. I‐20 showed low inhibition (< 50%) against *E. coli* and *S. aureus*, while I‐21 exhibited lower inhibition (< 55%) against *E. coli* and *L. monocytogenes* than the other isolates. When comparing the inhibition rates of the isolates against *E. coli*, the highest were observed in I‐3, I‐5, I‐6, I‐34 and I‐36, while the lowest were observed in I‐20 and I‐21. I‐5, I‐34 and I‐36 demonstrated the highest inhibition rates against *L. monocytogenes*, followed by I‐3, I‐6 and I‐20, with the lowest inhibition rate observed in I‐21. Against *S. aureus*, the highest inhibition rates were observed in I‐3, I‐5, I‐6, I‐21, I‐34 and I‐36, while the lowest inhibition rate was observed in I‐20.

### 3.5 16S rRNA Sequencing of Selected LAB Isolates

Using the 16S rRNA gene sequence, isolates of I‐3, I‐21 and I‐36 (GenBank accession numbers: PV345696, PV345694 and PV345692, respectively) exhibited 100% similarity to the reference sequence of *P. pentosaceus*, while isolates I‐5, I‐6 and I‐34 showed 100% similarity to the reference sequence of *L. plantarum* (GenBank accession numbers: PV345698, PV345700 and PV345699, respectively). The bacterial species were determined by comparing the obtained 16S rRNA nucleotide sequences with sequences in the NCBI GenBank database. The Nucleotide‐Nucleotide BLAST program available on the National Center for Biotechnology Information's accessible website (www.ncbi.nlm.nih.gov) was used to identify the bacterial species.

## 4 Discussion and Conclusion

LAB, due to its positive effects on human health and its role in fermentation, hold great importance in the food industry and have been safely used in food for many years. These organisms are utilized in various ways, including food production, disease treatment and the production of macromolecules, enzymes and metabolites. The LAB isolates obtained from traditional artisanal foods in this study demonstrated remarkable antimicrobial and antibiofilm activities against the tested foodborne pathogens. Using the unneutralized CFS in food biopreservation provides a more accurate and realistic representation of the total innate inhibitory potential of LAB strains isolated from conventional fermented matrices, making them potential bio‐protective agents (Yazgan et al. 2021). In the present study, evaluation of LAB's CFS without pH neutralization showed that the observed inhibitory effects were not solely due to the production of specific bacteriocins or targeted bioactive peptides. These activities are thought to result primarily from the synergistic effects of organic acids, including lactic acid, and other secreted secondary metabolites (Falqueto et al. 2026; Kim et al. 2026). The acidic environment created by lactic acid production is, in fact, the primary mechanism by which these isolates naturally exhibit protective and antibiofilm effects in real food environments (Falqueto et al. 2026; Singh et al., 2025).

It is widely known that LAB strains inhibit target pathogens by increasing cell membrane permeability through bacteriocin production and hydrogen peroxide formation, as well as by acidifying the environment (Darbandi et al. 2022; Alakomi et al. 2000). In the present study, the seven isolates showed a pattern that aligns with the selective antimicrobial patterns reported by Akpınar et al. (2011). While Akpınar et al. observed broad‐spectrum activity, isolates in this study showed a more specialized inhibitory profile, primarily targeting Gram‐positive indicators. This selective activity can be attributed to the diversity of LAB strains (Stupar et al. 2021). The antimicrobial activity of LAB is highly strain‐dependent and often governed by the production of various metabolites, such as organic acids and bacteriocins (Parada et al. 2007). Furthermore, the fact that pathogens such as *L. monocytogenes* are susceptible to the antibacterial activity of LAB, while it is ineffective against *E. coli*, suggests that antimicrobial compounds may have specific targets in the cell wall of Gram‐positive bacteria (Zhydzetski et al. 2024).

In the present study, LAB isolates demonstrated antimicrobial activity against *L. monocytogenes*, with inhibition zone diameters ranging from 8 to 11 mm, and slightly lower diameters (8–10 mm) against *B. cereus*. Consistent with the present results, various researchers have documented the broad‐spectrum antimicrobial potential of food‐borne LAB. For instance, Gülseren (2012) demonstrated that strains isolated from boza – including *Streptococcus salivarius, L. acidophilus, L. bulgaricus* and *L. lactis* – effectively inhibited a wide range of pathogens such as *B. subtilis, S. aureus, B. laterosporus, E. coli, B. cereus, B. brevis, B. circulans, B. coagulans* and *P. aeruginosa*. In line with the present results, Ertekin and Çon (2014) isolated 26 LAB from diverse traditional fermented foods and reported inhibition zones of 7.3–10.2 mm against *L. monocytogenes* and *E. faecium*. Furthermore, Pinto et al. (2020) confirmed that *Pediococcus* and *Lactobacillus* species isolated from food products exhibited antimicrobial activity against *S. aureus*, *E. coli*, *B. cereus* and *L. monocytogenes* at levels comparable to those reported in this work. These findings agree with previous reports indicating that LAB isolates tend to exhibit effective inhibitory effects against L. monocytogenes and B. cereus

Globally recognized regulatory bodies, including the European Food Safety Authority (EFSA) and the Clinical and Laboratory Standards Institute (CLSI), mostly provide standardized cutoff values for Minimum Inhibitory Concentration (MIC) tests. Still, specific disk diffusion breakpoints are not available for many LAB species (EFSA,^2012^).

Disk diffusion assays are widely recognized and accepted as preliminary phenotypic screening tools for evaluating the safety profiles of LAB isolates (Duche et al. 2023). In the absence of formal species‐specific disk diffusion guidelines, the resistance profiles were interpreted using criteria established in comparable previous studies (Tumbarski et al. 2021). Therefore, these findings provide an initial phenotypic evaluation of the safety profiles of local strains isolated from conventional matrices. Future studies should include genotypic evaluation and MIC determinations to provide a definitive, comprehensive assessment of antibiotic resistance.

In the present study, all seven LAB isolates revealed high susceptibility to Chloramphenicol and Ampicillin (zone diameters >16 mm) and intermediate susceptibility to gentamicin (7–16 mm). These results align with the established literature, which generally characterizes LAB as highly susceptible to beta‐lactams and chloramphenicol (D'Aimmo et al. 2007).

In a study by Liu et al. (2017), a similar susceptibility to Chloramphenicol and Ampicillin was reported among 41 LAB isolates, evaluated using disk diffusion and E‐test methods. Likewise, Arık (2018) reported a high susceptibility rate (77.5%) to Ampicillin among LAB strains recovered from various food matrices, reinforcing the conservation of these susceptibility patterns across different food‐borne isolates. In another study, Flórez et al. (2016) isolated a total of 34 strains belonging to *Leuconostoc mesenteroides* (18), *Leuconostoc citreum* (11), *Leuconostoc lactis* (2), *Weissella hellenica* (2) and *Leuconostoc carnosum* from milk and dairy products to determine their antibiotic resistance. They identified atypical resistance among their isolates to kanamycin (17 strains), tetracycline, erythromycin (two strains each), as well as clindamycin, virginiamycin, ciprofloxacin and rifampicin (one strain each). Concurrently, Gad et al. (2014) reported that the majority of their lactic acid isolates obtained from milk and dairy products were resistant to Vancomycin (40.6%) and Streptomycin (17.4%). In agreement with the current results, a large proportion of isolated LAB were resistant to ciprofloxacin, trimethoprim‐sulfamethoxazole and oxacillin. These findings are also supported by the literature, which indicates that *Lactiplantibacillus* and *Pediococcus* species possess intrinsic resistance to fluoroquinolones, folate pathway inhibitors and some beta‐lactams (Yin et al. 2025; Hummel et al. 2007; Ammor et al. 2007).

In recent years, the role of bacteriocins produced by LAB in combating biofilms as antibiofilm agents has attracted attention, particularly for eliminating pathogenic bacteria that form biofilms (Mathur et al. 2018; Duraisamy et al. 2020). In the food industry, biofilm formation typically begins with the attachment and proliferation of bacteria to organic matter accumulated on equipment, utensils and surfaces used in production and processing lines. The multilayered structure and exopolysaccharide matrix formed by microorganisms in biofilms make it difficult for antimicrobial agents to penetrate and reach the inner layers. This resilient structure enables microorganisms to withstand osmotic stress, toxic compounds, disinfectants and antibiotics (Tumbarski et al. 2021). The resistance of bacterial biofilms to antimicrobials necessitates the development of new methods to control biofilms formed by pathogens (Kumar and Anand 1998). It is well known that foodborne pathogens such as *Salmonella* spp., *L. monocytogenes* and *S. aureus* exhibit a high capacity for biofilm formation (Giaouris et al. 2014). In the present study, the antibiofilm effects of cell‐free supernatants from isolated LAB against *E. coli*, *L. monocytogenes* and *S. aureus* were determined using a 96‐well microplate method. The CFS of five out of seven LAB isolates (I‐3, I‐5, I‐6, I‐34 and I‐36) demonstrated exceptional antibiofilm efficacy (85% to 100%) inhibition against *E. coli*, *L. monocytogenes* and *S. aureus*. Conversely, isolates I‐20 and I‐21 displayed lower, strain‐specific inhibitory capacities (less than 50% and less than 55%, respectively), particularly against *E. coli* and *L. monocytogenes*. Notably, the highest biofilm eradication (100%) against *L. monocytogenes* was achieved with isolates I‐5, I‐34 and I‐36. Supporting these results, Kıran et al. (2021) reported that *L. plantarum* CFE demonstrated 90% antibiofilm activity against *L. monocytogenes*. Furthermore, Masebe and Thantsha (2022) confirmed that CFS from various LAB strains exhibited high antibiofilm activity against *L. monocytogenes* strains on polyvinyl chloride (PVC) and stainless‐steel surfaces. The strong antibiofilm profiles of LAB in the present study suggest that they may have high potential for use as natural biological control formulations to eliminate pathogen matrices in food processing environments. However, this strong antibiofilm activity seen in these isolates may be due to a synergistic effect of specific antibiofilm mechanisms, such as the low pH resulting from the accumulation of organic acids present in unneutralized CFS, which strongly restricts the growth of target pathogens such as *S. aureus* (Ammor et al. 2007; Hummel et al. 2007). Therefore, the decrease in biofilm biomass observed in this study may be partly due to the use of unneutralized CFS, which may inhibit viable planktonic cells that would otherwise initiate biofilm formation. To deeply investigate these effects, future studies incorporating neutralized CFS as an additional experimental control would be highly beneficial. However, from a food safety and preservation perspective, the overall efficacy demonstrated by CFS strains indicates strong potential to control the vegetative growth and biofilm formation of undesirable microorganisms under natural physiological conditions (Mani‐López et al. 2022).

16S rRNA sequencing is a widely used method for identifying and classifying LAB. This method compares the variable and conserved regions of the 16S rRNA gene to infer phylogenetic relationships among bacterial species (Johnson et al. 2019). In the present study, 6 LAB strains isolated from traditional artisanal dairy and meat products via 16S rRNA sequencing were identified as *P. pentosaceus* and *L. plantarum*. Parallel to these findings, Vanniyasingam et al. (2019) characterized the probiotic properties of eight LAB isolates obtained from cheddar cheese, yogurt and cow's milk. Based on probiotic properties, the isolated M6 strain was identified as *L. plantarum* CIP 103151 through 16S rDNA sequence analysis. Similarly, Pinto et al. (2020) examined 280 LAB isolated from various food products for their antimicrobial activities against foodborne pathogens and evaluated the probiotic potential of the bacteriocin‐producing isolates. Using 16S rRNA gene sequence analysis, they identified six *P. pentosaceus* and one *L. plantarum* strains among their seven bacteriocin‐producing LAB isolates, which strongly corroborates the species distribution observed in the present study. Furthermore, Kamiloğlu (2022) identified five *L. plantarum* strains isolated from fermented sausages in Turkey using 16S rRNA sequencing to evaluate their functional and technological properties. In a broader geographic screening, Pavli et al. (2016) isolated 47 different LAB strains from traditional fermented dairy and meat products and identified them using 16S rRNA gene sequencing. Those researchers stated that all strains showed 100% similarity to reference sequences and met the established probiotic criteria. Additionally, Mahajan et al. (2017) utilized 16S rRNA gene sequencing to successfully identify isolated LAB at the species level, classifying a prominent isolate as *L. plantarum*. Collectively, these literature reports confirm that *P. pentosaceus* and *L. plantarum* are naturally occurring, dominant and technologically important species in conventional fermentation ecosystems (Rahayu 2003).

In conclusion, 35 LAB strains were isolated from traditional artisanal dairy and meat products and selected based on their antimicrobial, antibiofilm and antibiotic susceptibility. The six most efficient strains were subjected to 16S rRNA sequencing, and I‐3, I‐21 and I‐36 were identified as *P. pentosaceus*, while I‐5, I‐6 and I‐34 were identified as *L. plantarum*. These strains exhibit promising preliminary in vitro functional properties, including antimicrobial activity and biofilm inhibition, and may have potential use in the production of functional foods. However, these strains require further research on cytotoxicity, animal model trials and comprehensive genomic safety profiling to ensure rigorous validation for human safety.

## Author Contributions

**Erdi Şen**: investigation, writing – review and editing. **Neslihan Öztürk**: investigation, writing – review and editing. **Erhan Keyvan**: writing – review and editing, writing – original draft. **Hatice Ahu Kahraman**: conceptualization, investigation, methodology, writing – original draft, supervision, writing – review and editing.

## Funding

This research has been supported within the content of the project no 0763‐YL‐21 ***/*** 2017K12‐41003 by Burdur Mehmet Akif Ersoy University Scientific Research Projects Unit.

## Conflicts of Interest

The authors declare no conflicts of interest.

## Data Availability

The data supporting this study's findings are available from the corresponding author upon reasonable request.
